# Association between the apoptotic effect of Cabazitaxel and its pro-oxidant efficacy on the redox adaptation mechanisms in prostate cancer cells with different resistance phenotypes

**DOI:** 10.1080/15384047.2024.2329368

**Published:** 2024-03-14

**Authors:** Isil Ezgi Eryilmaz, Unal Egeli, Gulsah Cecener

**Affiliations:** Faculty of Medicine, Medical Biology Department, Bursa Uludag University, Bursa, Turkey

**Keywords:** Cabazitaxel, prostate cancer, redox adaptation, Nrf-2, NF-κB, HIF-1α

## Abstract

Redox adaptation causes poor prognosis by adapting cancer cells to excessive oxidative stress. Previously, we introduced an oxidative stress-resistant metastatic prostate cancer (mPC) model (LNCaP-HPR) that redox adaptation reduced the effect of Cabazitaxel (Cab), the last taxane-derivative for metastatic castration-resistant PC (mCRPC). Whereas, we investigated for the first time whether there is an association between the altered apoptotic effect and pro-oxidant efficacy of Cab on the redox adaptation in PC cells with different phenotypes, including LNCaP mPC, LNCaP-HPR, C4–2 mCRPC, and RWPE-1 cells. Cab was shown pro-oxidant efficacy proportionally with the apoptotic effect, more prominent in the less aggressive LNCaP cells, by increasing the endogenous ROS, mitochondrial damage, and inhibiting nuclear ROS scavengers, p-Nrf2 and HIF-1α. However, the pro-oxidant and apoptotic effect was lower in the LNCaP-HPR and C4–2 cells, indicating that the drug sensitivity of the cells adapted to survive with more ROS was reduced via altered regulation of redox adaptation. Additionally, unlike LNCaP, Cab caused an increase in the p-NF-κB activation, suggesting that the p-NF-κB might accompany maintaining survival with the increased ROS in the aggressive PC cells. Moreover, the cytotoxic and apoptotic effects of Cab were less on RWPE-1 cells compared to LNCaP but were closer to those on the more aggressive LNCaP-HPR and C4–2 cells, except for the changing pro-oxidant effect of Cab. Consequently, this study indicates the variable pro-oxidant effects of Cab on redox-sensitive proteins, which could be a target for improving Cab’s apoptotic effect more in aggressive PC cells.

## Graphical abstract

**Figure:**
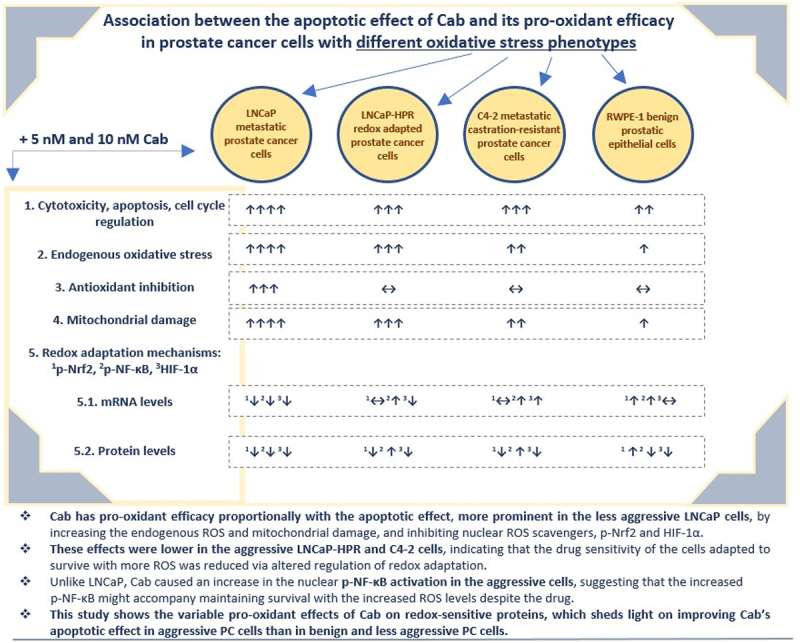

## Introduction

Cancer cells develop an adaptive process characterized by increased reactive oxygen species (ROS) levels and upregulation of cellular antioxidant capacity, called redox adaptation.^1^ As an acquired and dynamic feature, redox adaptation is associated with surviving under excessive ROS, inflammation, metastasis, and chemoresistance.^2–4^ Additionally, disruption of redox adaptation is a selective cancer treatment strategy via pro-oxidant agents that increase the endogenous ROS from tumorigenic to cytotoxic levels or decrease antioxidant capacity.^4,5^ Thus, understanding the role of redox adaptation for targeting cancer cells using pro-oxidants with antitumoral function helps to improve more selective therapy strategies. However, little is known about the pro-oxidant properties of novel chemotherapeutics.

Prostate cancer (PC) is the first in new cases and the second in all cancer deaths.^6^ Taxane-based chemotherapeutics such as Docetaxel (Doc) or the newest semi-synthetic derivative, Cabazitaxel (Cab), are effectively used for the treatment of metastatic (mPC) and/or metastatic castration-resistant PC (mCRPC).^7^ However, these agents are insufficient in the later mCRPC stages. The main reason for this failure is acquired drug resistance in cancer cells.^8,9^ Moreover, some studies have recently reported that endogenous ROS and the capacity to cope with this affect the treatment response of cancer cells.^10,11^ Thus, redox adaptation can alter the drug sensitivity, as we previously reported the reduced apoptotic effect of Cab in the first introduced mPC redox adaptation model.^12^ We showed that the process is mainly driven by the increase of ROS tolerance of the cells due to some biological differences between the oxidative stress-resistant and sensitive ones, such as the upregulation of the redox adaptation modulators, including nuclear factor erythroid-2-related factor-2 (Nrf-2), nuclear factor kappa-light-chain-enhancer of activated B cells (NF-κB) and hypoxia-inducible factor-1α (HIF-1α). Consequently, the Cab sensitivity of redox-adapted mPC (LNCaP-HPR) cells decreased compared to the sensitive ones.^12^

In the literature, previous studies reported that induction of ROS production can modulate the cytotoxic effect of taxane-based chemotherapeutics on cancer cells.^13,14^ Moreover, one study revealed that Cab showed significantly higher cytotoxic efficacy than Doc in human CRPC cells, accompanied by elevated Cab-induced ROS production, suggesting that Cab is an advantageous agent with more pro-oxidant effect than Doc. This study also noted that Cab could overcome Doc resistance, showing significantly higher cytotoxic and pro-oxidant effects than Doc on the resistant C4-2AT6 cells by inhibiting the expression of the antioxidant enzyme sestrin-3 (SESN3).^15^ However, the pro-oxidant effect of Cab and its related mechanistic action need further investigations due to a poor understanding of the association between Cab’s apoptotic effect and its pro-oxidant efficacy on the redox adaptation mechanisms.

Therefore, the present study hypothesizes whether the apoptotic effect of Cab is changing based on the oxidative stress tolerance and redox adaptation mechanisms of PC cells. Thus, for the first time, we comparatively investigated the pro-oxidant efficacy of Cab on redox adaptation mechanisms and association with the apoptotic effect in PC cells with different resistance phenotypes, including LNCaP mPC, redox-adapted LNCaP-HPR, C4–2 mCRPC, and RWPE-1 benign prostate cells.

## Results

### Cytotoxic effect of Cab

**Figure 1.**
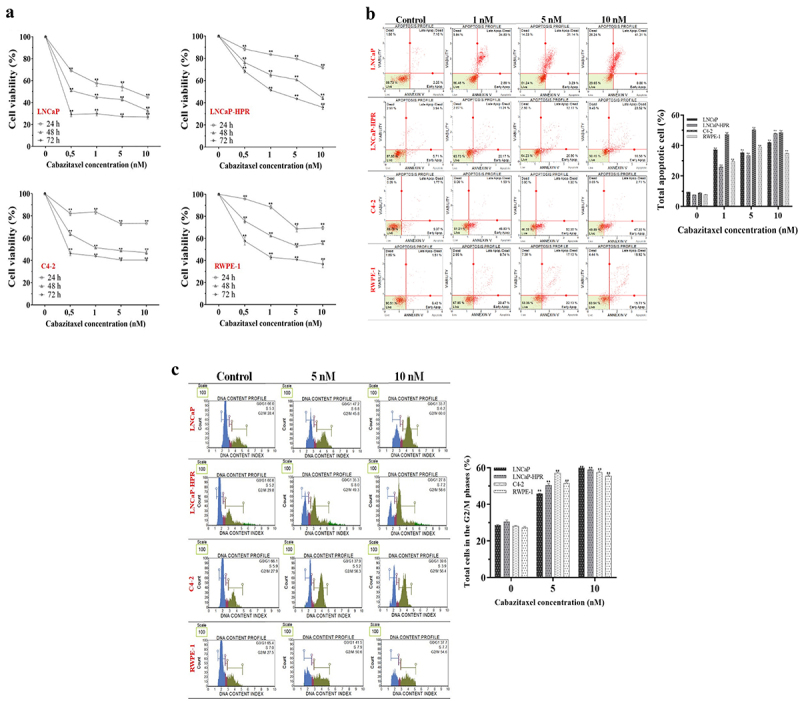
(a) Effects of increasing concentrations (0.5, 1, 5, and 10 nM) of Cab on the cell viability of Cab-treated cell lines for 24, 48, and 72 h (*n* = 6, **p < .01) (b) Annexin V assay reveals the apoptotic effect of Cab at the increasing concentrations as 1, 5 and 10 nM in Cab-treated cell lines for 48 h (*n* = 3, **p < .01) (c) The PI-based cell cycle assay indicates the effect of different Cab concentrations on the G2/M cycle arrest of Cab-treated cells for 48 h (*n* = 3, **p < .01). Effects of increasing Cab concentrations are plotted on the horizontal axis for the annexin V and cell cycle assays. At the same time, analysis results for different cell lines (the names of the cells are highlighted in red) are arranged vertically; *p*-values were calculated by one-way ANOVA and Tukey’s multiple comparisons tests in GraphPad Prism 9.0 (La Jolla, CA, USA). In the graphs, **p < .01 indicates significant differences compared to the control group of each cell line. Cab, Cabazitaxel; control, non-Cab-treated group.

The cytotoxicity of Cab was significantly higher in LNCaP among the PC cells (Figure 1a). The viabilities were found as 59.7%, 55.4%, and 45.7% at 1, 5, and 10 nM in the 24-h Cab-treated LNCaP cells, respectively (*p* < .01). However, the viability of LNCaP-HPR or C4–2 did not reach ~ 50% at 24 h. For 48 h, the viabilities were detected as 64.9%, 61.7%, 44.1% (*p* < .01), and 51.4%, 49.7%, 48.9% (*p* < .01) at 1, 5, and 10 nM in the Cab-treated LNCaP-HPR and C4–2, respectively. At 10 nM, LNCaP viability decreased to 32.6% (*p* < .01). Furthermore, on RWPE-1 cells, 5 and 10 nM Cab caused a significant viability reduction to 71.0%, 70.2% and, 55.7%, 61.1% for 24 and 48 h, respectively (*p* < .01). However, for 72 h, RWPE-1 viability was 40.7% for 5 nM and 41.0% for 10 nM doses. Therefore, 48 h was chosen as an optimal Cab treatment time due to the selective cytotoxicity on PC cells.

### Apoptotic and cell cycle regulatory effects of Cab

In LNCaP cells, the late apoptosis rate was significantly increased (*p* < .01) to 31.1% and 41.3% after 5 and 10 nM, respectively. Moreover, the dead cells were higher in 48-h Cab-treated LNCaP compared to LNCaP-HPR and C4–2 cells (Figure 1b). Total apoptotic LNCaP-HPR cells were 32.9% and 40% after 5 and 10 nM Cab for 48 h, respectively (*p* < .01). Moreover, apoptotic death was significantly triggered after 48-h Cab treatment in C4–2 cells, and the apoptotic cells increased from 11.14% to 49.8% at 10 nM (Figure 1b). Thus, the apoptotic profile of 10 nM Cab-treated LNCaP-HPR was closer to that of C4–2, not parental LNCaP cells. Cab also significantly activated apoptosis in RWPE-1 cells with a percentage of 34.6% (*p* < .01) at 10 nM. The statistics are represented in the graph of Figure 1b.

Additionally, Cab caused a significant cell cycle arrest at the G2/M phases (Figure 1c). However, the arresting effect was detected mainly in LNCaP, increasing the cells at the G2/M phase from 28.4% to 45.8% and 60.0% (*p* < .01) at 5 and 10 nM, respectively. Moreover, Cab caused a similar effect at 10 nM in LNCaP-HPR and C4–2, increasing the cells at the G2/M from 29.8% to 58.6% (*p* < .01) and 27.9% to 56.4% (*p* < .01), respectively. The Cab also increased the RWPE-1 cells at G2/M from 27.5% to 50.4% (*p* < .01) and 54.6% (*p* < .01) at 5 and 10 nM, respectively. The statistics are represented in the graph of Figure 1c.

### Pro-oxidant effects of Cab

**Figure 2.**
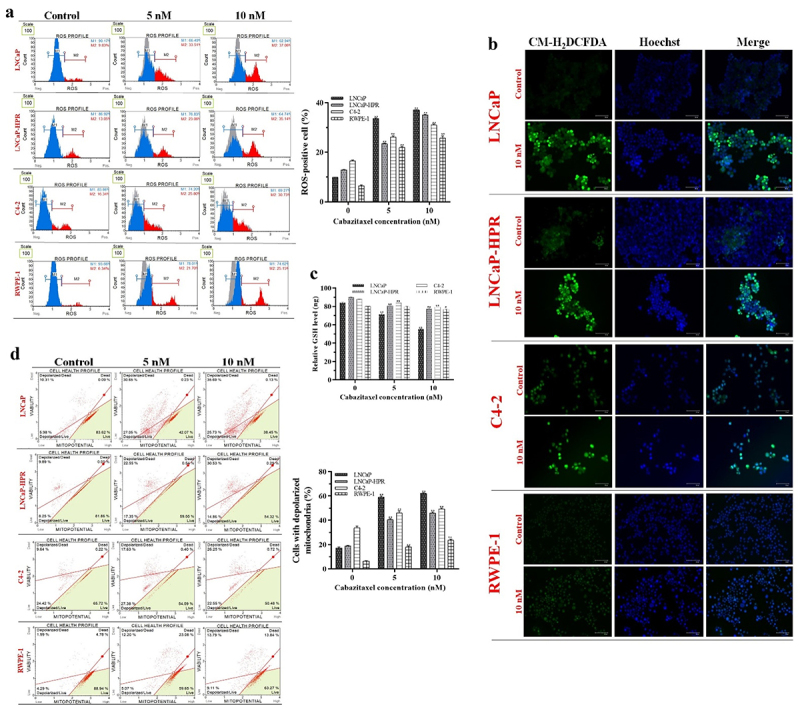
(a) Effects of 5 and 10 nM Cab on the ROS profile of 24 h-Cab treated cell lines (*n* = 3, **p < .01) (b) Morphological ROS analysis of 10 nM Cab indicates that the accumulation of H_2_O_2_ in the subcellular localization visualized by green fluorescence increase after Cab treatments in different cells. The merge images show the H_2_O_2_-positive cells with Hoechst dye for nuclear staining in the total cell population for each cell line (c) Effect of different Cab concentrations on the total GSH (ng) levels of each cell line (*n* = 3, *p < .05, **p < .01). The levels of total GSH as ng are calculated using the standard calibration curve with seven different standard concentrations: 0, 20, 40, 60, 80, 100, and 120 ng. Based on the OD values of these standards at 415 nm, the standard curve graph equation used to calculate the changes in the total GSH levels of the cells is y = 0.0231× + 0.8426 (d) Effects of 5 and 10 nM Cab on the mitochondrial membrane potential of Cab-treated cell lines for 24 h (*n* = 3, **p < .01). Effects of increasing Cab concentrations are plotted on the horizontal axis for the ROS and MitoPotential assays. At the same time, analysis results for different cell lines (the names of the cells are highlighted in red) are arranged vertically; *p*-values were calculated by one-way ANOVA and Tukey’s multiple comparisons tests in GraphPad Prism 9.0 (La Jolla, CA, USA). In the graphs, *p < .05, **p < .01 indicates significant differences compared to the control group of each cell line. Cab, Cabazitaxel; control, non-Cab-treated group; ROS, reactive oxygen species; GSH, glutathione; H_2_O_2_, hydrogen peroxide.

We first determined the optimum time, 24 h, for the most ROS-inducing effect of Cab before the most effective apoptotic one. We showed that the non-treated cells were ranked from lowest to highest as RWPE-1 (6.34%), LNCaP (9.83%), LNCaP-HPR (13.05%), and C4–2 (16.34%), based on endogenous ROS. While 5 and 10 nM Cab significantly increased the ROS-positive cells to 33.51% and 37.06% in LNCaP (*p* < .01), the ROS-positive LNCaP-HPR and C4–2 cells were significantly (*p* < .01) increased to 23.06% and 35.15%; 25.80% and 30.73%, respectively (Figure 2a). Additionally, Cab caused a significant increase in the endogenous ROS in RWPE-1, and the ROS-positive cells were increased to 21.70% and 25.15% at 5 and 10 nM, respectively (*p* < .01). The statistics are shown in the graph of Figure 2a.

Morphological ROS analysis first indicated that LNCaP-HPR cells’ endogenous ROS levels were more than those of LNCaP and more resemble non-treated C4–2 cells (Figure 2b). Then, 10 nM Cab caused more accumulation of H_2_O_2_ in PC cells than in RWPE-1. After Cab treatment, at most, ROS accumulation was observed in LNCaP, LNCaP-HPR, C4–2, and RWPE-1 cells, respectively (Figure 2b).

Moreover, Cab reduced the total GSH levels of the cells in a dose-dependent manner (Figure 2c), which is more prominent in LNCaP compared to LNCaP-HPR and C4–2. Cab decreased the 84.09 ng total GSH levels of LNCaP cells to 71.51 ng and 55.29 ng at 5 and 10 nM, respectively (*p* < .01). Besides, the same treatments caused a less reduction in the total GSH levels of LNCaP-HPR, from 90.07 ng to 80.76 ng and 77.31 ng (*p* < .01), respectively. Moreover, in the C4–2 cells, 5 and 10 nM Cab treatments decreased the 87.83 ng total GSH levels to 83.84 ng and 79.15 ng (*p* < .01), respectively. In RWPE-1 cells, while 5 nM Cab did not induce a significant change, 10 nM reduced the total GSH level from 80.09 ng to 76.79 ng (*p* < .05).

### The effect of Cab on mitochondrial membrane potential

The disruption of mitochondrial membrane potential is an indicator of ROS-dependent cell death. The depolarized mitochondria rates in the non-treated cells were 16.29%, 18.14%, 34.06%, and 6.28% in LNCaP, LNCaP-HPR, C4–2, and RWPE-1 cells. 5 nM Cab caused a significant increase in the rate of 57.7%, 39.9%, 45.0%, and 17.27% in the same cell order (*p* < .01). Moreover, 10 nM Cab significantly increased the membrane depolarization to 61.42%, 45.39%, 48.8%, and 22.9% in LNCaP, LNCaP-HPR, C4–2, and RWPE-1 cells, respectively (*p* < .01) (Figure 2d). Thus, we showed that Cab caused more mitochondrial damage in LNCaP, which has the lowest mitochondrial damage without Cab, than in LNCaP-HPR, C4–2, and RWPE-1 cells. The statistics are represented in the graph of Figure 2d.

### The effect of Cab on the mRNA levels of the redox-sensitive transcription factors and antioxidant enzymes

**Figure 3.**
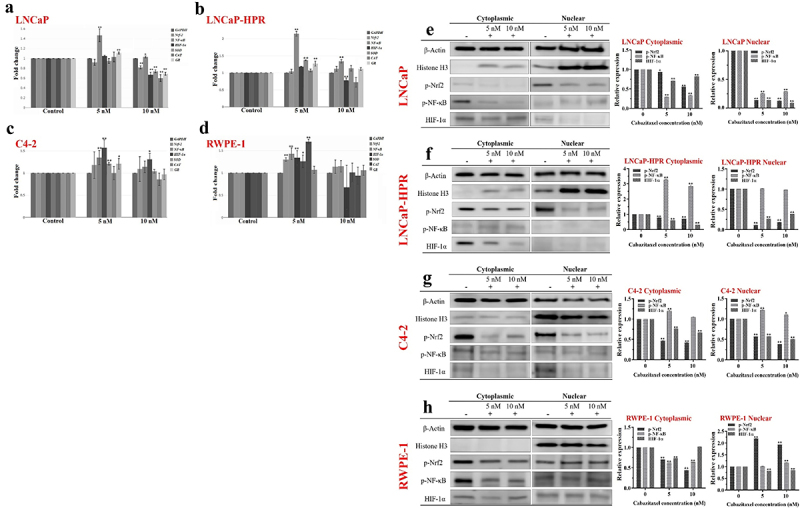
Effect of 5 and 10 nM Cab on the mRNA levels of the redox-sensitive transcription factors and antioxidant enzymes in (a) LNCaP (b) LNCaP-HPR (c) C4–2 (d) RWPE-1 cells (*n* = 3, *p < .05, **p < .01). *GAPDH* was used as a housekeeping gene to normalize mRNA expression levels of the indicated genes. The fold changes of mRNAs and *p*-values were calculated using the 2^–ΔΔCt^ method in a web‑based tool at https://dataanalysis2.qiagen.com/pcr to analyze gene expression differences between the control and Cab-treated groups. Effect of increasing Cab concentrations on the active protein levels of the redox-sensitive transcription factors at cytoplasmic and nuclear compartments of Cab-treated (e) LNCaP (f) LNCaP-HPR (g) C4–2 (h) RWPE-1 cell lines (*n* = 3, *p < .05, **p < .01). Relative protein expression at cytoplasmic fractions was determined using β-actin as an internal control. Moreover, histone H3 was used as a housekeeping protein to calculate the relative expression levels of the nuclear active forms of the indicated proteins. *P*-values were calculated by one-way ANOVA and Tukey’s multiple comparisons tests in GraphPad Prism 9.0 (La Jolla, CA, USA). In the relative protein expression graphs, *p < .05, **p < .01 indicates significant differences compared to the control group of each cell line for cytoplasmic and nuclear fractions.

In LNCaP cells, Cab caused a 1.47-fold (*p* < .01) increase in the *NF-κB* levels. However, at 10 nM, *NF-κB* was decreased to nearly the control level. 10 nM Cab also significantly decreased the levels of *Nrf-2* and *HIF-1α* to 1.22-fold and 1.50-fold, respectively (*p* < .01). Moreover, the levels of antioxidant genes, *SOD, CAT*, and *GR*, were decreased by 1.35-fold, 1.67-fold, and 1.46-fold at 10 nM in LNCaP, respectively (*p* < .01) (Figure 3a). Besides, in LNCaP-HPR, 5 nM Cab caused a significant increase by 2.15-fold, 1.37-fold, and 1.27-fold in the *NF-κB*, *SOD*, and *GR* mRNAs, respectively (*p* < .01). In the same cells, while the *NF-κB* was significantly increased by 1.34-fold (*p* < .01), the *HIF-1α* was decreased by 1.27-fold (*p* < .01) at 10 nM (Figure 3b). In C4–2, 5 nM caused a significant increase in the levels of *NF-κB*, *HIF-1α*, *SOD*, and *GR* to 1.35-fold (*p* < .01), 1.57-fold (*p* < .01), 1.21-fold (*p* < .01), and 1.20-fold (*p* < .05), respectively. Moreover, 10 nM Cab increased the expression of *HIF-1α* to 1.31-fold (*p* < .05) in C4–2 (Figure 3c). In RWPE-1, 5 nM caused an increase in the mRNAs of *Nrf-2*, *NF-κB*, and *HIF-1α*, to 1.30-fold, 1.43-fold, and 1.34-fold, respectively (*p* < .01). Moreover, the levels of *SOD* and *CAT* significantly increased by 1.26-fold (*p* < .05) and 1.70-fold (*p* < .01), respectively, in the 5 nM Cab treated-RWPE-1 (Figure 3d).

### The effect of Cab on the protein levels of the active redox-sensitive transcription factors

Cab caused significant decreases in p-Nrf2, p-NF-κB, and HIF-1α levels in LNCaP. Mainly, the nuclear levels were significantly decreased by 7.5-fold, 4.0-fold, and 7.4-fold at 5 nM, respectively (*p* < .01). Moreover, at 10 nM, the nuclear levels of the p-Nrf2, p-NF-κB, and HIF-1α were significantly decreased by 8.3-fold, 3.5-fold, and 12.0-fold, respectively (*p* < .01) (Figure 3e). In LNCaP-HPR, 5 and 10 nM Cab significantly increased the cytoplasmic expression of p-NF-κB by 3.24-fold and 2.78-fold, respectively (*p* < .01) (Figure 3f). However, no significant change was detected in the nuclear p-NF-κB. Moreover, while the nuclear p-Nrf2 and HIF-1α were decreased by 9.0-fold and 3.8-fold at 5 nM (*p* < .01), the fold changes were 5.4-fold and 2.6-fold at 10 nM in LNCaP-HPR cells, respectively (*p* < .01). In C4–2, p-Nrf2 and HIF-1α levels were significantly decreased in both fractions (Figure 3g). 10 nM Cab reduced considerably by 2.6-fold and 1.98-fold in the nuclear p-Nrf2 and HIF-1α levels, respectively (*p* < .01). However, 5 and 10 nM Cab triggered significant increases in the cytoplasmic and nuclear p-NF-κB of C4–2 cells to 1.20-fold (*p* < .01) and 1.16-fold (*p* < .05) (Figure 3g). Interestingly, in the Cab-treated RWPE-1, the cytoplasmic levels of p-Nrf2, p-NF-κB, and HIF-1α were significantly decreased; however, the nuclear p-Nrf2 was increased considerably by 2.18-fold and 1.92-fold at 5 and 10 nM, respectively (*p* < .01). Moreover, 10 nM Cab triggered a slight increase in the nuclear p-NF-κB level of RWPE-1 (Figure 3h).

### The effect of Cab on the subcellular localizations of the redox-sensitive transcription factors

**Figure 4.**
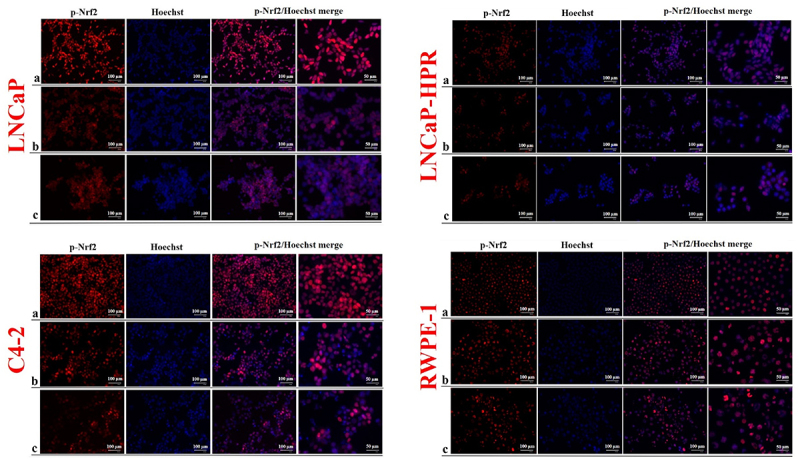
Immunofluorescence analysis shows the effect of the increasing Cab concentrations on the p-Nrf2 expression and subcellular localization in the Cab-treated cell lines. On the horizontal axis of the microphotograph group for each cell line, (a) control, (b) 5 nM, and (c) 10 nM Cab. For each group, six independent images were obtained. In the groups for each cell line, the last two columns are the merged images for the indicated protein and nuclear staining with Hoechst dye. The scale bar represents 100 µm at 20X magnification. However, the microphotographs in the last column were obtained as larger images using a 50 µm scale bar at 20X magnification.

The level of p-Nrf2, distinctly localized in the nucleus, was more dramatically decreased after Cab treatments in LNCaP than in LNCaP-HPR and C4–2 cells in a dose-dependent manner (Figure 4). Besides, the decrease of nuclear p-Nrf2 was slighter in LNCaP-HPR than in LNCaP cells; however, 5 nM Cab caused a further reduction in the p-Nrf2 nuclear localization in LNCaP-HPR. In addition, the p-Nrf2 level in the nucleus of C4–2 cells decreased after increasing doses of Cab. However, nuclear expression of the p-Nrf2 was increased at 5 and 10 nM Cab in RWPE-1 cells (Figure 4).

**Figure 5.**
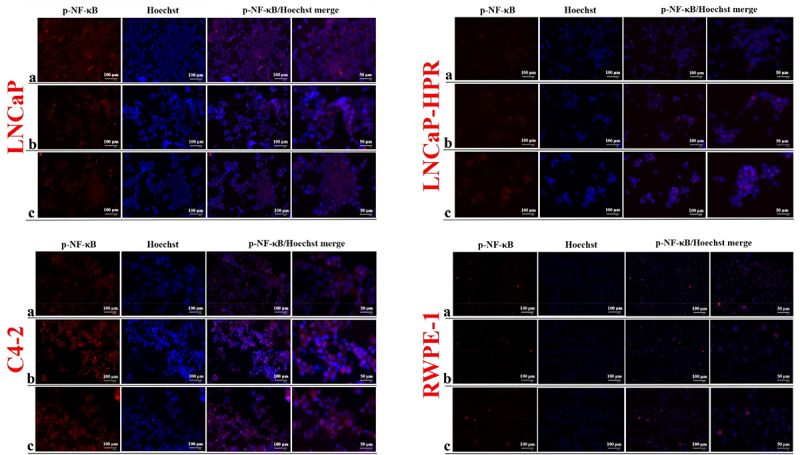
Immunofluorescence analysis shows the effect of the increasing Cab concentrations on the p-NF-κB expression and subcellular localization in the Cab-treated cell lines. On the horizontal axis of the microphotograph group for each cell line, (a) control, (b) 5 nM, and (c) 10 nM cab. For each group, six independent images were obtained. In the groups for each cell line, the last two columns are the merged images for the indicated protein and nuclear staining with Hoechst dye. The scale bar represents 100 µm at 20X magnification. However, the microphotographs in the last column were obtained as larger images using a 50 µm scale bar at 20X magnification.

Moreover, p-NF-κB expression in both localizations decreased dose-dependently in LNCaP (Figure 5). However, in LNCaP-HPR, p-NF-κB was increased in the cytoplasm after Cab treatment, especially at 5 nM. Similarly, in C4–2 cells, Cab treatment caused an increase of cytoplasmic and nuclear p-NF-κB. In RWPE-1 cells, a slight decrease was also detected in the nuclear p-NF-κB; however, this was not as remarkable as in the LNCaP cells (Figure 5).

**Figure 6.**
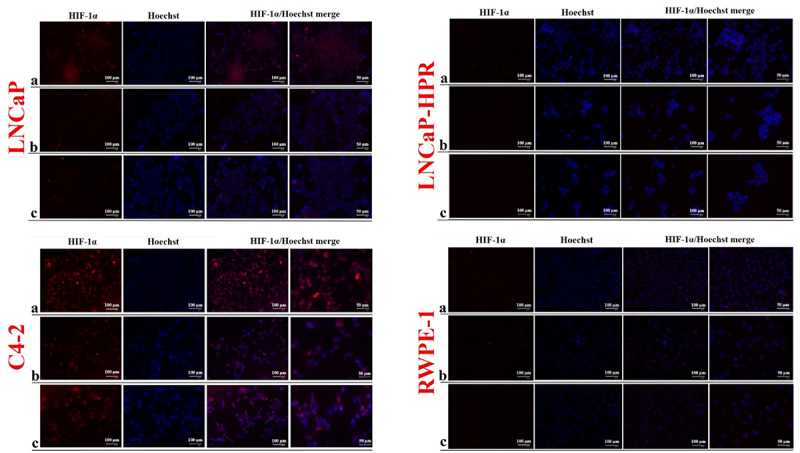
Immunofluorescence analysis shows the effect of the increasing Cab concentrations on the HIF-1α expression and subcellular localization in the Cab-treated cell lines. On the horizontal axis of the microphotograph group for each cell line, (a) control, (b) 5 nM, and (c) 10 nM cab. For each group, six independent images were obtained. In the groups for each cell line, the last two columns are the merged images for the indicated protein and nuclear staining with Hoechst dye. The scale bar represents 100 µm at 20X magnification. However, the microphotographs in the last column were obtained as larger images using a 50 µm scale bar at 20X magnification.

Finally, we observed a decrease in the cytoplasmic and nuclear expression of HIF-1α after the increasing doses of LNCaP (Figure 6). However, due to the low basal expression, the HIF-1α decrease in the subcellular localizations was not clearly observed in LNCaP-HPR cells. Moreover, in C4–2, HIF-1α was mainly detected in the nucleus and decreased by increasing Cab doses (Figure 6). In RWPE-1, basal expression of HIF-1α was relatively low; however, the lower expression of HIF-1α was in the Cab-treated group.

## Discussion

In the present study, we investigated whether the apoptotic effect of Cab is changing based on the oxidative stress tolerance and redox adaptation mechanisms of PC cells. Oxidative stress tolerance in cancer cells leads to progressive gains that complicate treatment, such as increased metastatic potential and ROS-mediated changes reducing chemotherapeutics sensitivity.^2,10,11^ We previously have shown that the apoptotic effect of Cab was decreased in the redox-adapted LNCaP-HPR cells compared to sensitive LNCaP cells to exogenous ROS. Moreover, after the adaptation, the Cab response of the redox-adapted cells also resembled those of more aggressive C4–2 mCRPC cells, unlike LNCaP.^12^ This time, the association between the apoptotic effect of Cab and its pro-oxidant efficacy on the redox adaptation mechanisms in PC cells has been evaluated. Moreover, being reported that the oxidative stress states of PC cells were directly proportional to the aggressive characteristics,^16^ we used four cells with different aggressivity, including LNCaP mPC, redox-adapted LNCaP-HPR mPC, C4–2 mCRPC, and RWPE-1 benign cells. The results show that, while the most apoptotic effect of Cab was observed in the less aggressive LNCaP mPC cells, the lower was detected in LNCaP-HPR and C4–2 mCRPC cells. RWPE-1 viability was also at most 60% at 48 h, the earliest time Cab was effective against LNCaP-HPR and C4–2. Therefore, we investigated whether the apoptotic effect is affected by the endogenous ROS-related alterations in the cells.

We determined that Cab has significant pro-oxidant effects by increasing ROS and inducing apoptosis with mitochondrial damage in a dose-dependent manner, mainly in the less aggressive LNCaP mPC cells. These effects were lower in the other PC and RWPE-1 cells. In addition, when the endogenous ROS of the non-treated cells was compared, the RWPE-1 and LNCaP mPC cells had the least; LNCaP-HPR and C4–2 mCRPC cells had higher levels, compatible with the flow cytometric and morphological results, supporting the literature revealing that endogenous ROS levels were changing proportionally based on the aggressiveness.^16^ Moreover, Cab increased the ROS levels and mitochondrial damage in proportion to the aggressive properties of PC cells, which were determined to have higher pro-oxidant effects in the LNCaP mPC than in the LNCaP-HPR and C4–2 mCRPC cells. The cells were ranked as LNCaP˃LNCaP-HPR˃C4–2 depending on the mitochondrial defect after Cab, indicating that the redox adaptation affects the cells’ sensitivity to pro-oxidant agents. Thus, Cab caused a higher increase in the ROS levels in the less aggressive PC cells but had lower effects in more aggressive ones and less triggered apoptosis mediated by mitochondrial dysfunction. Similar effects were also seen in RWPE-1, and the effects of the drug that impaired mitochondria health were proportional to the apoptotic activity. In the literature, Cab has been reported to cause a higher increase in endogenous ROS in the Doc-resistant C4-2AT6 mCRPC cells than Doc.^15^ These results indicate that the decreased Doc sensitivity led to a decrease in the pro-oxidant effects of the drug, in which the cells were resistant. Therefore, the gaining aggressive properties of PC cells affect the taxane response, and this is most likely related to drug-targeted changes in the endogenous oxidative stress, as in our results regarding the changes in the Cab response of PC cells with different phenotypes. Moreover, we showed that Cab has low effects on GSH in PC cells, except LNCaP. However, unlike other cells, in LNCaP, decreased GSH levels might be associated with the triggering of more ROS by Cab, which is independent of the direct effect on antioxidant inhibition because, despite Cab, at least 85% of the total GSH was preserved in LNCaP-HPR, C4–2, and RWPE-1 cells. The absence of differences in the total GSH between Doc-resistant and sensitive PC cells suggests that taxane activity does not affect GSH metabolism.^15^ Thus, we concluded that Cab did not show direct pro-oxidant effects in PC cells via antioxidant inhibition but increased endogenous ROS.

Cancer cells adapt to the increased ROS during redox adaptation by upregulating the redox-sensitive transcription factors such as Nrf2, NF-κB, and HIF-1α.^17^ This offers undeniable advantages to cancer cells, including survival under stressful conditions, metastasis, and chemoresistance.^3–5^ Therefore, disruption of the redox adaptation therapeutically targets the cancer cells via pro-oxidant therapy.^18^

Our results indicated that the inhibitory effect of Cab on the genes encoding redox-sensitive transcription factors and antioxidant defense enzymes was not stable and relatively low, showing the transcriptional fold changes after the Cab treatment generally ranged under 2.0. However, *NF-κB* mRNA increased after Cab treatment in all cells, 2.15-fold in LNCaP-HPR. Especially for the redox-sensitive transcription factors, gene expression is regulated at various stages, most commonly at the post-transcriptional and the post-translational levels by protein-protein interaction or phosphorylation, which affects the active protein levels and results in incompatible mRNA/protein regulation.^19,20^ Thus, no significantly detectable changes could be found in the mRNA levels of these genes. However, Cab treatment significantly regulated these proteins’ active levels and subcellular localization.

As shown in the protein expression results, Cab significantly affected the levels and the subcellular localizations of the redox-sensitive transcription factors and inhibited the nuclear p-Nrf2 in all PC cells, mainly in LNCaP. The main redox-sensitive transcription factor, Nrf-2, is activated to provide cellular defense against oxidative stress with many targets, including antioxidant proteins, and is commonly upregulated in advanced tumors.^19,21^ Nrf-2 activation is mainly controlled via Ser40 phosphorylation by the protein kinase C (PKC) and subsequent disruption of the Nrf2-Keap1 association.^19^ So far, the regulatory effect of Cab on the Nrf-2 signaling has not been investigated. However, in Doc-resistant mCRPC cells, not Doc but Cab has been shown to increase ROS via inhibition of SESN3, an oxidative stress resistance protein, by blocking the Nrf2-Keap1 association.^15^ Thus, the higher sensitivity of the cells to Cab has been reported to involve ROS production by Cab via inhibiting the antioxidant SESN3 levels.^15^ Moreover, the changes in the levels of various kinases, including PKC, are among the mechanisms involved in Cab response and long-term Cab-resistance of mCRPC cells.^22^ As consistent with the findings, p-Nrf2 levels significantly decreased in all Cab-treated PC cells. However, this effect was more prominent in LNCaP mPC compared to LNCaP-HPR and C4–2 mCRPC cells. In contrast, in RWPE-1 cells, p-Nrf2 slight activation after Cab might be associated with the hypoactivation of PKC or the stability of the endogenous cytostatic ROS despite Cab in the cells with benign characteristics.

In the literature, the effect of Cab on the redox-sensitive transcription factor HIF-1α is unknown. We found that, especially in LNCaP and LNCaP-HPR cells, Cab reduced the nuclear HIF-1α, whose activity is generally controlled at the post-transcriptional level.^21^ These inhibitory effects varied proportionally with the PC cells’ endogenous ROS levels; the least was detected in RWPE-1. Therefore, we indicate that the inhibition of p-Nrf2 and HIF-1α mediated the pro-oxidant effects of Cab on PC cells, and these effects were more prominent in the less aggressive LNCaP mPC cells with the lowest endogenous ROS in non-treated conditions, unlike LNCaP-HPR and C4–2 mCRPC cells with more aggressive phenotypes.

When the effects of Cab on p-NF-κB were examined, there was a significant decrease in the *NF-κB* gene expression and in the protein levels at cytoplasmic and nuclear localizations in LNCaP cells. However, the mRNA of NF-κB and the levels of p-NF-κB were significantly increased after Cab treatment in more aggressive LNCaP-HPR and C4–2 cells. Two studies, one carried out by our team, revealed that NF-κB activation was increased in response to Cab in pancreatic cancer and mCRPC cells, and this activation was consistent with the pro-apoptotic activity of the drug.^23,24^ Moreover, Cab efficacy increased when combined with NF-κB inhibitors in advanced pancreatic cancer.^23^ Interestingly, we showed for the first time that Cab inhibited p-NF-κB in LNCaP cells, on which more apoptotic effect was detected. However, Cab increased the level of p-NF-κB in the less responsive PC cells. These results suggest that after the acquisition of oxidative stress resistance, similar to the response of C4–2 mCRPC cells, LNCaP-HPR cells increased the level of p-NF-κB after Cab treatment, indicating that NF-κB inhibition might be a therapeutic strategy for enhancing Cab sensitivity in aggressive PC cells. Moreover, stress oncoproteins have been reported to be important in sensitizing chemoresistant mCRPC cells to taxanes.^25^ Thus, we suggest synergistically disrupting redox adaptation with the agents targeting p-NF-κB is most likely to improve the apoptotic effect of Cab in aggressive PC cells via selective inhibitors of the indicated pathway. Further studies should focus on additional findings to support these results using inhibitors of the pathways and/or rescue experiments in conditions with or without cytokine treatments.

Consequently, we evaluated the effect of Cab on the redox adaptation mechanisms for the first time. Cab, the last taxane derivative for treating mCRPC, has a pro-oxidant impact proportionally with the apoptotic effect in PC cells with different resistance phenotypes. Cab displays the pro-oxidant effect by increasing the endogenous ROS levels, mitochondrial membrane damage, and inhibiting redox scavenger factors p-Nrf2 and HIF-1α, which are more prominent in the less aggressive LNCaP mPC cells. However, the drug’s pro-oxidant efficacy and apoptotic effect were decreased in the more aggressive LNCaP-HPR and C4–2 cells, indicating that the Cab sensitivity of the cells, which are adapted to survive with more endogenous oxidative stress, was reduced due to the Cab-triggered and altered regulation of the redox-sensitive transcription factors. The drug has pronounced apoptotic and pro-oxidant effects on the redox-sensitive transcription factors in LNCaP cells. However, this mechanism was less effective in the redox-adapted LNCaP-HPR and C4–2 mCRPC cells. Additionally, NF-κB activation in response to Cab in these aggressive cells indicates that Cab’s apoptotic and pro-oxidant effects were most likely reduced due to maintaining the high ROS levels accompanied by the increased p-NF-κB in the cells. Since the cells adapt to survive with more oxidative stress, Cab’s pro-oxidant effect at the same treatment conditions is insufficient to reach the ROS level in these cells to cytotoxic levels as in LNCaP cells. As a result, this study clarifies that the effects of Cab on redox adaptation mechanisms contribute to improving the apoptotic effect of the drug in more aggressive PC and mCRPC cells.

## Materials and methods

### Chemicals

Cab (99.87%) was purchased from Selleckchem (Houston, TX, USA). Fetal bovine serum (FBS), RPMI-1640 medium, antibiotic‑antimycotic, Trypsin‑EDTA, CM-H_2_DCFDA, Qubit Protein Assay, Keratinocyte SFM, Reverse-transcription kit and expression assays were purchased from Thermo Fisher Scientific (Waltham, MA, USA). Bovine Serum Albumin (BSA), DMEM-low glucose, H_2_O_2,_ and Hoechst were obtained from Sigma-Aldrich (Darmstadt, Germany). WST-1 and Glutathione Assay were purchased from BioVision (San Francisco, CA, USA). Muse® Annexin‑V Dead Cell, Cell Cycle, Oxidative Stress, and MitoPotential kits were obtained from Luminex (Austin, TX, USA). RNA isolation kit was obtained from Omega Bio‑Tek (Norcross, GA, USA). Cytoplasmic and nuclear protein extraction kit was purchased from Boster (Pleasanton, CA, USA). SDS Gels, Nitrocellulose papers, Tris/Glycine/SDS, and Tris/Glycine were obtained from Bio-Rad (Hercules, CA, ABD). The primary antibodies, p-Nrf2 (ab76026), p-NF-κB (ab86299), HIF-1α (ab51608), β-Actin (ab228001), IgG HRP-conjugated secondary antibody (ab6721), IgG secondary antibody Alexa Fluor-594 (ab150080), Protein ladder (5–245 kDa), protease-phosphatase inhibitor, normal goat serum and DAPI Mounting media were obtained from Abcam (Cambridge, UK). Histone H3 (D1H2) primary antibody, Tris buffered-saline with Tween 20 (TBST), and Nonfat dry milk were purchased from Cell Signaling Technology (Danvers, MA, USA).

### Cell culture

We used three PC cells with different phenotypes and RWPE-1 benign prostate cells. The human mPC LNCaP (CRL‑1740™) and prostatic epithelial RWPE-1 (CRL-11609™) cells were purchased from the American Type Culture Collection. The C4–2 mCRPC cells were kindly provided by Dr. Thalmann from the Urology Department, University of Bern. LNCaP-H_2_O_2_ resistant-(HPR) cells were established by continuously applying 50 μM H_2_O_2_ to parental LNCaP cells as we reported previously,^12^ and the H_2_O_2_ concentration to which the cells adapt was added to the complete media. The culture conditions were previously reported for LNCaP, LNCaP-HPR, and C4–2 cells.^12^ RWPE-1 cells were cultured using 1X keratinocyte SFM. The cultures were maintained at 37°C and 5% CO_2_ in a humid incubator (Panasonic, Japan). Confluent cells were passaged using Trypsin-EDTA. All cells were checked for contamination using the MycoProbe Detection Kit (R&D Systems, MN, USA) and Hoechst staining.

### Cab treatment and cytotoxicity test

The Cab was prepared in DMSO as a 5 mM stock and stored at − 80°C. Before each experiment, drug solutions were freshly diluted to 50 nM with the growth medium of each cell and applied at concentrations of 0.5, 1, 5, and 10 nM. The DMSO ratio was kept below 0.002%. Briefly, the cells were seeded in 96-well plates at appropriate densities and treated with Cab for 24–72 h. At the end, WST-1 was added to the wells and incubated for 2 h. Then, relative cell viability as percent values compared to the non-treated controls was determined by measuring color intensity in six replicates at 450 nm in a microplate reader (Berthold Technologies, Bad Wildbad, Germany).

### Cell death and cell cycle analysis

To determine apoptotic and cell cycle effects of Cab, Muse® Annexin‑V and Dead Cell and Cell Cycle Kits were used, respectively. The cells were seeded in 6-well plates at 1-5×10^5^/ml and 5 × 10^5^-1×10^6^/ml for sequential assays and treated with Cab for 48 h. After washing twice with PBS, cells were stained with Annexin-V and incubated in the dark for 30 min. For cell cycle analysis, cells were fixed using 70% cold ethanol for 3 h, then stained with dye for 30 min. The cells were analyzed in three replicates using a Muse® Cell Analyzer (Merck Millipore, Darmstadt, Germany).

### Flow cytometric oxidative stress analysis

Based on the Muse® Oxidative Stress kit, the cells were prepared in 1X assay buffer at 1 × 10^6^/ml and incubated with 1:100 dye solution for 30 min at 37°C. After incubation, stained cells were analyzed to detect ROS-positive percentages in three replicates using a Muse® Cell Analyzer (Merck Millipore, Darmstadt, Germany).

### Morphological oxidative stress analysis

The changes in the endogenous ROS of the cells were morphologically analyzed with CM-H_2_DCFDA staining. For fluorescence imaging, the cells were washed twice with PBS and stained with 10 µM CM-H_2_DCFDA for 45 min in growth conditions. After checking the green fluorescence showing ROS-positivity, the cells were fixed immediately with 4% paraformaldehyde for 20 min. Finally, nucleus staining was performed with 10 µg/ml Hoechst; then, the cells were visualized under a fluorescence microscope (Thermo Fisher, Waltham, MA, USA) with six independent images.

### Determination of cellular GSH level

The GSH levels of the cells were determined using a colorimetric assay. The treated cells were collected at 4°C and washed once with PBS. Then, the pellets were lysed in the cold buffer for 10 min. After destroying cellular proteins by adding sulfosalicylic acid, supernatants were taken in equal volumes into a 96-well plate, and a reaction was constructed. After 15 min, the absorbances were measured in triplicate at 415 nm in a microplate reader (Berthold Technologies, Bad Wildbad, Germany). The concentrations of total GSH in the sample solutions were determined using the total glutathione standard calibration curve that we obtained with seven different standard concentrations: 0, 20, 40, 60, 80, 100, and 120 ng.

### Mitochondrial membrane potential analysis

To determine the mitochondrial health, the cells with a density of 1-5×10^5^/ml were washed once with PBS and stained with 95 μl mitopotential dye for 20 min in growth conditions. After incubation, 5 μl 7-AAD dye was used to stain death cells. Then, analysis was performed in three replicates using a Muse® Cell Analyzer (Merck Millipore, Darmstadt, Germany).

### Gene expression

We performed gene expression analysis for the redox-sensitive transcription factors and antioxidant enzymes to evaluate the pro-oxidant effect of Cab on the redox adaptation mechanisms at the RNA level. Briefly, 1 × 10^6^ cells were seeded in *T*-25 flasks for the treatment, and then total RNA samples were isolated from the cells. After converting from 100 ng RNAs to cDNAs, expression analysis was performed as previously reported in triplicate for *Nrf-2*, *NF-κB*, *HIF-1α*, *SOD1*, *CAT*, and *GSR*.^12^

### Protein expression

Western Blot analysis was used to determine how the active levels of redox-sensitive transcription factors were regulated after Cab treatment. A total of 1 × 10^6^ cells were seeded in *T*-25 flasks. After treatments, the cytoplasmic and nuclear-fractionated proteins were isolated from the cells. The concentrations of proteins were determined using a Qubit 2.0 Fluorometer (Thermo Fisher, Waltham, MA, USA). A total of 30 µg samples were loaded into the gels and run for 1–2 h at 100 V. After, the membrane was blocked with 5% milk or BSA in TBST and incubated with primary antibodies, p-Nrf2 (1:20000), p-NF-κB (1:5000), HIF-1α (1:1000), Histone H3 (1:2000) and β-Actin (1:10000), overnight at 4°C with agitation. Then, secondary antibody (1:5000) incubation was performed for 1 h. After washing three times with TBST, chemiluminescence detection was performed. The protein bands were viewable in a C-DiGit® Blot Scanner (LI-COR, Lincoln, NE, USA). Finally, the Image*J* program (National Institutes of Health, Bethesda, MD, USA) was used to detect band intensities used in the statistics.

### Immunofluorescence staining

The cells were seeded into 6-well plates at a density of 5 × 10^5^/well and treated with Cab. The cells were washed once with cold PBS and then fixed with 4% paraformaldehyde for 15 min. After washing three times with cold PBS, cells were permeabilized with 0.1% Triton X-100 and then blocked with 1% BSA and 5% goat serum for 1 h. Then, the cells were subjected to p-Nrf2 (1:100), p-NF-κB (1:500), and HIF-1α (1:100) antibodies for 1 h with agitation. After washing with PBS three times, secondary antibody (1:500) incubation was performed for 1 h. Finally, nuclear staining was applied with 10 µg/ml Hoechst, and then the cells were visualized under a fluorescence microscope (Thermo Fisher, Waltham, MA, USA) with six independent images.

### Statistics

The GraphPad Prism 9.0 (La Jolla, CA, USA) was used to determine the statistical meaning of the results. The treated groups were compared with the control group using one-way ANOVA with an appropriate post hoc test. The statistics of the gene expression results were tested with the 2^–ΔΔCt^ method in a web‑based tool at https://dataanalysis2.qiagen.com/pcr. A *p*-value less than 0.05 was considered statistically significant.

## Data Availability

The data that support the findings of this study are available from the corresponding author, [author initials], upon reasonable request.
